# Health Outcomes in Women Victims of Intimate Partner Violence: A 20-Year Real-World Study

**DOI:** 10.3390/ijerph192417035

**Published:** 2022-12-19

**Authors:** Maria Clemente-Teixeira, Teresa Magalhães, Joana Barrocas, Ricardo Jorge Dinis-Oliveira, Tiago Taveira-Gomes

**Affiliations:** 1 Department of Public Health and Forensic Sciences, and Medical Education, Faculty of Medicine, University of Porto, 4200-319 Porto, Portugal; 2 Center for Health Technology and Services Research (CINTESIS), 4200-450 Porto, Portugal; 3 MTG Research and Development Lab, 4200-604 Porto, Portugal; 4 TOXRUN–Toxicology Research Unit, University Institute of Health Sciences, Advanced Polytechnic and University Cooperative (CESPU), CRL, 4585-116 Gandra, Portugal; 5 USF Caravela, Local Healthcare Unit of Matosinhos, Lagoa Street, 4460-352 Senhora da Hora, Portugal; 6 Abel Salazar Biomedical Sciences Institute, University of Porto, 4050-313 Porto, Portugal; 7 UCIBIO-REQUIMTE, Laboratory of Toxicology, Department of Biological Sciences, Faculty of Pharmacy, University of Porto, 4050-313 Porto, Portugal; 8 Department of Community Medicine, Information and Decision in Health, Faculty of Medicine, University of Porto, 4200-319 Porto, Portugal; 9 Faculty of Health Sciences, University Fernando Pessoa (FCS-UFP), 4249-004 Porto, Portugal

**Keywords:** intimate partner violence, female, health risk behaviours, health outcomes, health care diagnosis

## Abstract

Intimate partner violence is characterized by violent actions against a person perpetrated by his or her former or current partner, regardless of cohabitation. It most frequently affects women, and one of its most relevant outcomes is the health problems associated with the experience of repeated violence. Thus, the main objective of this study is to analyse the prevalence of health problems among women for whom there was a medical suspicion of being victims of intimate partner violence. The specific objectives are to analyse the prevalence of (a) health risk behaviours; (b) traumatic injuries and intoxications; (c) mental health conditions; and (d) somatic diseases. We conducted a real-world, retrospective, observational, cross-sectional and multicentric study based on secondary data analyses of electronic health records and health care register data in patients of the Local Healthcare Unit of Matosinhos (between 2001 and 2021). The identified data were extracted from electronic health records corresponding to the Health Insurance Portability and Accountability Act Safe Harbor Standard. Information was obtained considering the International Classification of Diseases, the International Classification of Primary Care, and the Anatomical Therapeutic Chemical Classification System, as well as clinical notes (according to previously defined keywords). Considering all information sources, 1676 cases were obtained. This number means that just 2% of the women observed at this health care unit were suspected of being victims of intimate partner violence, which is far from the known statistics. However, we found much higher rates of all health risk behaviours, trauma and intoxication cases, mental health conditions, and somatic disorders we looked for, when compared to the general population. Early detection of these cases is mandatory to prevent or minimize their related health outcomes.

## 1. Introduction

The World Health Organization (WHO) considers domestic violence, including intimate partner violence (IPV), as a serious public health problem worldwide and has included it in the codes of the International Classification of Diseases [1]: ICD 9-995.80, 995.81, 995.82, 995.83, 995.84, 995.85, and ICD 10 T76. This organization admits that this special type of disease, which can manifest itself in very diverse ways, is associated with relevant morbidity and mortality in the short, medium, and long term [2]. Domestic violence is a huge problem with social roots, found in all types of families, regardless of the sex, age, and familiar (or similar) relation between the involved persons (victim and offender).

IPV is characterized by violent actions (physical, emotional, psychological, and sexual, among others) against a person, perpetrated by his or her former or current partner, regardless of cohabitation, and occurs in all cultures and countries [3]. IPV most frequently affects women [4]. A report by WHO states that 15% to 71% of all women suffer from physical and/or sexual abuse from an intimate partner at least once during their lifetime [5]. These situations of continued violence, apart from depriving the victim of his or her rights and freedom, result in a wide and severe diversity of consequences: physical, psychological, sexual, reproductive, relational, educational, professional, economic, and those regarding quality of life and dignity. In the most critical cases, it could lead to temporary or permanent disability or even death. This violence is also responsible for victims’ feelings of shame, fear, and even guilt, which often explains the lack of disclosing cases [6] and facilitates his or her control by the aggressor, leading to a perception of isolation, helplessness, and incapacity to react [7].

Regarding the health problems associated with IPV, the short-term consequences include acute traumatic body injuries and acute psychological damage [8,9]; these require immediate attention of professionals and the entire community. If the case is not reported and treated early and adequately, there is a risk of the perpetuation of the violence cycle [10], which is associated with the risk of violence growing in frequency and severity, exacerbating the health outcomes that may surge in the medium and long term. These outcomes are health risk behaviours associated with the risk of trauma, intoxication, and physical and mental disorders. They have a cumulative effect, considering the frequency and type of violence suffered [11].

Long-term morbidity is due to the influence of traumatic/toxic stress on the normal functioning of various systems linked to body homeostasis (e.g., neurological, immunological, and endocrine systems). This favours the development of dysfunctions that manifest themselves at different levels of organic functioning due to, among other problems, chronic hyperactivation of the hypothalamic–pituitary–adrenal axis, which influences the inflammatory response and causes hormonal changes, such as increased production of cortisol, decreased expression of its receptors, and increased corticotrophin-releasing factor [12,13,14]. The dysfunction of this axis is very much linked to the development of diseases, both mental and physical, due not only to its dysregulation but also because it can act as a trigger for some genetically programmed and latent diseases in the absence of toxic stress [13,14]. This dysfunction may also contribute to a proinflammatory state that favours the development of physical and psychological illnesses, such as depression or rheumatoid arthritis [13,14,15].

Health risk behaviours are more frequent in female victims than in the general population and may include [16] (a) inadequate diets; (b) abusive substance consumption (e.g., drugs, psychotropic substances, alcohol, and tobacco) [12,13]; (c) physical inactivity; (d) sexual risk behaviours (e.g., multiple partners, unprotected sexual intercourse); and (e) self-destructive behaviours (e.g., self-inflicted injuries and suicide attempts) [17,18].

Concerning mental health, on a medium- and long-term basis, higher rates of disorders are also found in female victims, namely (a) cognitive disturbances; (b) anxiety disorders; (c) hypervigilance; (d) phobias; (e) panic attacks; (f) depression; (g) posttraumatic stress disorder; (h) sleep disorders; (i) eating disorders; (j) body image disruption; (k) low self-esteem; (l) chronic pain [14]; (m) menstrual cycle disorders; (n) suicide ideation and attempts [17]; (o) homicide ideation; and (p) psychosomatic diseases [19].

Regarding physical health, there is a risk of chronic diseases, some of which are relatively common in the general population but may have a higher prevalence in IPV victims. Chronic/toxic stress related to violent experiences can be a trigger for health problems. However, this relationship is rarely identified by physicians. Some examples of these diseases are [20] (a) metabolic diseases (e.g., obesity, dyslipidaemia, type 2 diabetes); (b) cardiocerebrovascular diseases (e.g., atherosclerosis, arterial hypertension, acute myocardial infarction, stroke); (c) respiratory diseases (e.g., chronic obstructive pulmonary disease); (d) inflammatory diseases (e.g., rheumatoid arthritis, lupus, and asthma); and (e) neoplastic diseases. In sexual and reproductive health, we can more frequently find the following conditions [16]: (a) inflammatory pelvic disease; (b) urinary infections; (c) sexually transmitted infections; (d) unwanted pregnancy; (e) spontaneous and elective abortion; and (f) pregnancy complications.

Therefore, health care professionals have a crucial role in detecting this kind of violence and identifying and communicating the cases to authorities every time they have a reasonable suspicion of IPV [9]. In this context, the role of the hospital emergency departments and primary health care is particularly relevant because they represent an important gateway for these victims, and thus they have enormous importance regarding violence detection.

In Portugal, domestic violence is a public crime (Article 152° of the Portuguese Penal Code), and IPV is one of the most studied and identified types. In the 2020 annual report of *Associação Portuguesa de Apoio à Vítima* (APAV, Lisbon, Portugal), among the crimes against people, domestic violence crimes were the most common (72.6%), with 70.4% of them related to female victims and males being the most common perpetrators as partners or ex-partners [11].

An emergency department is a place where victims often go for the treatment of injuries, since it functions for 24 h every day [2]. A study revealed that IPV female victims resort to these departments three times more frequently than other people [2]. Moreover, through the holistic approach of primary health care, general practitioners can contribute to identifying these cases, since they know the victim’s household as well as the relationships and dynamics between the family elements. Additionally, general practitioners have a privileged type of doctor–patient relationship that allows them to obtain information related to these events. Thus, a local health care unit (LHU), as organized in Portugal (i.e., including both emergency departments and primary health care), can be a relevant source for studying these cases in specific geographic areas. The health care sector represents a key role in this approach, not only for early case detection and health care but also for supporting the victim, building an empathic relationship, validating concerns, and referring her to the competent authorities [21,22].

Preventing, detecting, and intervening in IPV cases requires a transdisciplinary approach, since there is a frequent need for the contribution of clinical medicine and psychology, forensic medicine, social services, police services, and public prosecution services, which ideally should work in an integrated way. This system still does not exist in Portugal. There has been a lack of political and other institutions to prepare an integrated national system for intervention on behalf of victims of violence.

The main goal of this study is to analyse the prevalence of health problems in women whom physicians from an LHU suspected to be victims of IPV. The secondary objectives are to analyse the prevalence, in this group, of (a) health risk behaviours; (b) trauma injuries and intoxications; (c) mental disorders; and (d) somatic diseases.

## 2. Materials and Methods

We conducted a real-world, retrospective, observational, cross-sectional, and multicentric study (14 primary care centres and 1 hospital). It was based on secondary data analyses of electronic health records (EHRs) and health care register data for patients of one of the eight Portuguese local health care units. The study was performed at the local health care unit of Matosinhos (LHUM), a northern Portuguese centre that provides primary, secondary, and tertiary health care. LHUM serves an urban population of approximately 172,669 inhabitants. As this is a study of databases with an eligible population in the hundreds of thousands, the application of informed consent is not feasible (subparagraphs (i) and (j) of Article 9° of the Portuguese General Data Protection Regulation). Data access for analysis was granted after approval by the Ethics Committee and data protection officer of the LHUM—approval codes No. 14/CES/JAS of 11-02-2022 (original) and No. 04/CLPSI/2022 of 02-03-2022 (original). All data processing and analysis were performed exclusively by analytic programs developed for this purpose and sent for execution on LHUM servers. No data were extracted outside LHUM, and no direct access by the researchers took place. As an additional degree of security, processed data were de-identified by the LHUM Information Technology Department prior to the analytic code execution according to the Health Insurance Portability and Accountability Act (HIPAA) safe harbour standard.

### 2.1. Participants

We considered the following inclusion criteria: (a) women; (b) aged 16 to 60 years old (the minimum age was assumed considering that at this age we can already find cases of dating violence [23] (the 60-year limit was considered to avoid confusion with many of the health problems associated with ageing); (c) suspected by an LHUM physician of being a victim of IPV (according to Article 144° of the Portuguese Penal Code, the physical, emotional, psychological or sexual violence, among others, perpetrated against a person, by his or her former or current partner, regardless of cohabitation); (d) part of the resident population served by LHUM; (e) with at least one clinical record entry in the past year; and (f) having at least one appointment with a primary care physician in the 3 years before the index date. The last data lock point was 20 March 2022 and included data collection from 1 January 2001 to 31 December 2021.

The results were analysed considering each source of information. Thus, 3 groups were defined: (a) G1, corresponding to the information resulting from codes and clinical notes (excluding the overlapping cases); (b) G2, including information resulting from codes only; and (c) G3, including information resulting from clinical notes only.

### 2.2. Variables

The following variables were considered, among others: (a) sex; (b) age; (c) health risk factors; (d) traumatic injuries and intoxications; (e) mental health disorders; (f) somatic diseases; and (g) follow-up at LHUM. Variables were defined using a group of keywords selected by the researchers (MC-T, TM). The keywords were later assessed by another researcher (TT-G) before inclusion in the analytic code for processing (RJD-O). The rationale for the keyword selection was based on the most common terms used to describe this kind of situation in the clinical setting of LHUM. Data regarding variables were classified using the (a) International Classification of Diseases (ICD-9 and ICD-10); (b) International Classification of Primary Care (ICPC-2); and (c) Anatomical Therapeutic Chemical Classification System (ATCCS).

The database contains the full records of (a) general practitioners’ visits; (b) emergency care; (c) prescription data; (d) hospital admissions; and (e) sick leave. As categorical variables we reported (a) smoking; (b) alcohol abuse; (c) drug abuse; (d) bone fractures; (e) bone dislocations; (f) open wounds; (g) superficial injuries; (h) crushing injuries; (i) burns; (j) intoxications; (k) headaches; (l) sleep disorders; (m) eating disorders; (n) poor health perception; (o) unspecified chronic pain; (p) memory disorders; (q) anxiety disorders; (r) major psychiatric disorders; (s) posttraumatic stress disorder; (t) suicidal ideation; (u) social deprivation; (v) sedative consumption; (w) anxiolytic consumption; (x) antidepressant consumption; (y) antipsychotic consumption; (z) obesity; (aa) hypercholesterolemia; (aa) metabolic syndrome; (bb) type 2 diabetes; (cc) nonalcoholic fatty liver disease; (dd) asthma; (ee) chronic obstructive pulmonary disease; (ff) hypertension; (gg) early heart disease; (hh) myocardial infarction; (jj) ischaemic stroke; (kk) haemorrhagic stroke; (ll) chronic kidney disease; (mm) sexually transmitted infections; (nn) preeclampsia; (oo) desired pregnancy; (pp) undesired pregnancy; (qq) natural abortion; (rr) voluntary abortion; (ss) inflammatory pelvic disease; (tt) urinary tract infection; (uu) chronic immune inflammatory disorder; (vv) cancer; and (ww) cervical cancer.

### 2.3. Data Sources/Measurement

The LHUM has one hospital (Pedro Hispano Hospital) and 14 primary care centres: 11 familiar health care units and 3 personalized health care units. In addition, LHUM also receives patients from other geographies and institutions to provide specific care needs. LHUM has 20 years’ worth of electronic health record (EHR) data containing complete information for every patient. Data were obtained from the computerized medical records of LHUM. All patients meeting the inclusion criteria were enrolled. All the data to be analysed in the study were already recorded in the database at the start of the study. No samples were taken, as we analysed the total patient population at LHUM that matched our eligibility selection.

### 2.4. Bias

The main source of bias for this study is omission bias. While this methodology ensures that no immediately relevant data were missed in the analysis, in IPV cases, besides the victim’s disclosure difficulties, care providers usually do not chart/code suspected cases. Thus, this study is subject to the risk of prevalence underestimation. To mitigate this bias, we used broad inclusion criteria and no exclusion criteria, employing a twofold approach (a) considering all relevant ICPC-2, ICD-9, ICD-10, and ATCC codes ever registered in all levels of care; and (b) considering all relevant keywords ever written by clinicians in the electronic health records. No other potential sources of bias were identified by the authors.

### 2.5. Statistical Methods

A descriptive analysis was performed without formal comparisons or other statistical analyses. For all variables, we report relative and absolute frequencies. For all variables that were computed with information aside from diagnosis codes, the percentage of null values (ø) was calculated (data not shown). To preserve anonymity, all frequencies of less than 5 cases are presented as <5 and the respective percentage.

## 3. Results

The values obtained for the numbers and rates of suspected victims of IPV are described in Table 1, considering the respective source of the information (G1, G2, and G3), which in G1 was 2.3% of the global female population of LHUM. During the 20 analysed years, the population of women from 16 to 60 years old and the population suspected of being victims of IPV (corresponding to the defined inclusion criteria) are described in Table 1. The median age of the general population was 41 years, and for the alleged IPV victims, it was 45, 48, and 42 years, considering G1, G2, and G3, respectively. Regarding the social conditions of the victims, poverty rates (Code Z01 of ICPC-2) of 3.6%, 6.3%, and 1.6% were found for G1, G2, and G3, respectively, while in the general population, it was 0.5%. The victims’ history of substance consumption, traumatic injuries and intoxications, mental health disorders, and somatic diseases are described in Table 2, Table 3, Table 4 and Table 5, respectively.

## 4. Discussion

Our results show that IPV victims present higher rates of health risk behaviours and comorbidities than the general population from the same context. To our knowledge, this is the first large-scale study of IPV conducted using population-level data extracted from Electronic Medical Records.

### 4.1. IPV and Health Risk Behaviours

It is known that women victims of IPV are at higher risk of engaging in risky behaviours, especially in cases where the violence started early in their lives (some from the moment of conception, being subjected after birth to abuses that constitute adverse childhood experiences, which have, with high probability, consequences for their future health [12]). These may be health risk behaviours or deviant behaviours, which may overlap [16]. Regarding health risk behaviours, these include inappropriate diet [16] and the increased tendency to (a) abuse substances [13,15,20,24] (e.g., medications, often anxiolytics and/or analgesics; drugs of abuse; alcohol; tobacco); (b) physical inactivity [16]; and (c) sexual risk behaviours [13,15,20] (e.g., multiple partners, unprotected intercourse).

Regarding substance consumption, we also found that health risk behaviours were higher in the alleged IPV population (G2) than in the general population: tobacco, alcohol, and drug consumption were 1.5, 6.5, and 13 times higher, respectively (Table 2). As we can see in Table 3, medication consumption is also higher. All of these behaviours, of which only a few examples have been given, contribute to the morbidity and mortality associated with IPV.

### 4.2. IPV, Traumatic Injuries, and Intoxications

The data analysed unequivocally show an increase in the rates of traumatic injuries and intoxications in alleged IPV cases when compared to the general population (Table 3). This is very relevant since, as the literature shows, women who are victims of IPV are at increased risk not only of abusive trauma [25] and intoxications [26] but also for accidental or self-inflicted injuries [13,15]. The obtained results do not allow for a differential diagnosis of the medicolegal aetiology of injuries and intoxications, so no further progress can be made in this discussion. However, superficial injuries are 2.2 times higher (G2) than in the general population, and it is known that they are also most frequent in IPV cases [27,28], which leads us to consider that many of these injuries may have been intentionally inflicted by an intimate partner. Regarding intoxications, they are also two times higher (G2), but here again, the medicolegal aetiology of the cases is unknown.

### 4.3. IPV and Mental Health Conditions

Mental health disorders in IPV cases in the short-long term may be related to the chronic stress experienced (psychosomatic manifestations or functional disorders, resulting from the somatization of anxiety) [29,30,31], such as (a) headaches (including migraines); (b) sleep and eating disorders; (c) odynophagia or pharyngeal ball sensation; (d) palpitations; (e) vague, nonspecific malaise complaints; (f) digestive complaints (e.g., nausea, nonspecific abdominal pain); (f) concentration and attention difficulties, with mild memory impairment and suicidal ideation; (g) back pain; and (h) various chronic pain syndromes or nonspecific pain. This whole process can evolve quickly, causing low self-esteem and negative self-concept (personal devaluation), which further aggravates the vulnerability (emotional fragility or dependence, with lack of trust in others) as well as the passivity of these victims [16]. In the medium to long term (sometimes even several years after the violence stops), these women develop more structured disorders. The consequences at a distance in time are especially related to the experience of traumatic violent situations, and this perspective is fundamental to understanding and intervening in this type of violence. The literature shows that comparing women who suffer this type of violence with women who do not suffer it, the first group presents higher levels of [19,29]: (a) cognitive disorders (concentration, attention, memory, repeated thoughts about violence, or cognitive distortion—disturbed thinking with misinterpretation of facts experienced); (b) anxiety disorders; (c) hypervigilance; (d) phobia; (e) panic attacks; (f) depression (sometimes with attempted or completed suicide, often associated with pregnancy and postpartum); (g) posttraumatic stress disorder; (h) sleep disorders (e.g., insomnia); (i) eating disorders (e.g., anorexia, bulimia); (j) body image changes; (k) low self-esteem; (l) chronic pain; (m) menstrual cycle disorders; (n) self-destructive behaviours [13,15] (e.g., self-inflicted injuries, suicidal ideation, suicide attempts); (o) homicidal ideation; and (p) substance dependence. In our study, some of these conditions were also found, including medication consumption (Table 4). We verified that by comparing alleged IPV victims (G2) with the general population, sleep disorders, unspecified chronic pain, anxiety disorders, and major psychiatric disorders increased 3.6, 2.4, 2.7, and 2.2 times, respectively. The medication consumption follows the previous results. Regarding anxiolytic consumption, we found that 87.5% of these women used these drugs (1.7 times more than the general population). The same is true for sedatives, antidepressants, and antipsychotics (2.0, 2.1, and 3.2 times higher than the general population, respectively). It should be noted that, contrary to expectations, suicidal ideation was coded in only 2.5% of the cases, which is far below what is reported in the literature [17]. Regardless, compared to the general population, the increase is 8.6 times greater. Regarding social deprivation, it was observed that this condition is 7.9 times more frequent in suspected IPV cases.

### 4.4. IPV and Somatic Health Conditions

Somatic health disorders in IPV cases may be related to the stressful experience of violence, which is almost always chronic, as mentioned before, manifesting themselves through symptoms or even well-established pathological presentations. These will likely be quite common, as clinical evidence is beginning to show [20], but their aetiology is not identifiable or is difficult to determine; thus, these situations end up being treated as general diseases, without careful attention to the reasons behind their origin. However, traumatic stress associated with violent experiences may cause disturbances in the body’s homeostasis by interfering with several systems, namely the neurological, immunological, and endocrine systems. Over time, various dysfunctions can arise that manifest themselves at different levels of organic functioning, such as (a) metabolic disorders [20,32] (e.g., obesity, dyslipidaemia, diabetes); (b) cardiocerebrovascular diseases [15,33,34] (e.g., atherosclerosis, hypertension, acute myocardial infarction, stroke); (c) respiratory disorders [20] (e.g., chronic obstructive pulmonary disease); (d) inflammatory disorders [35] (e.g., rheumatoid arthritis, lupus, asthma); and (e) neoplastic disorders [20]. There are also sexual and reproductive health consequences, such as (a) pelvic inflammatory disease; (b) urinary tract infections; (c) sexually transmitted infections; (d) sexual dysfunction; (e) unwanted pregnancies; (f) spontaneous or voluntary unsafe abortions; and (g) pregnancy complications. These consequences are directly related to forced contact, even within intimate relationships, and are also due to the inability to discuss with the aggressor the possibility of contraception, namely through the use of condoms, or with the late search for prenatal care in the case of pregnant women. IPV is especially serious and frequent during pregnancy. In addition to the risk of abortion or stillbirth, there is an increased possibility of problems for the baby, which may influence his or her future physical and mental health [16].

Table 4 describes the somatic disorders in the general LHUM population and in the women suspected of being victims of IPV. Considering G2, we found that all the analysed diseases and medical health conditions are more prevalent in women victims of IPV than in the general population (e.g., obesity, metabolic syndrome, type 2 diabetes, hypertension, early heart disease, myocardial infarction, ischaemic and haemorrhagic stroke, urinary tract infection, sexually transmitted infections, asthma, cancer). The only exception is for the desired pregnancy condition, which has an inferior rate, as expected, which somehow “validates” the obtained results.

### 4.5. The Care of Women Victims of IPV in a Local Health Care Unit

The organization and structure of Local Health Units in Portugal allows for a greater articulation of care through integrated responses. Thus, a greater articulation of care is possible through integrated responses in the respective region, benefiting from the proximity to the population, through the coordination of the services that operate there, including “safe houses” for women and respective children, when needed. In this way, they enhance service structures that are close to the people and integrated into local victim support services. Like all care levels in the NHS, these units are public and tend to be free of charge. In this sense, people with lower economic resources can use these services. Despite the excellent conditions of health organizations with the LHU typology to attend to these cases, we still find difficulties in the detection and recording of IPV suspicion by physicians. In fact, we verified that the IPV suspicion by physicians occurs for only 2.3% (G1) of the global female population. This rate is much lower than the known prevalence values for these cases recently reported in the literature [36]: 27% worldwide, 20% in Western Europe, and 18% in Portugal. This does not necessarily mean that physicians have not detected the cases; they may have just decided not to register and report them. However, it can also be explained by the detection difficulties, since many women victims do not disclose or even deny violence due to [6]: (a) persistent sociocultural issues that affect women’s perceptions about violent behaviour, leading some of them to still not identify themselves as victims; (b) the particular relationship of closeness and trust between the victim and the aggressor, based on emotional dependence and, sometimes, also on economic dependence; and (c) their submission and resignation to the control and violence perpetrated by the aggressor. In addition, in the cases where a record was made, comparing the results between G2 and G3, we find that alleged IPV cases are more often described using clinical codes (G2) than clinical notes (G3). This circumstance may be related to issues of medical confidentiality, which may create added difficulties for physicians who deal with these cases. Considering these results, we may state that there is still much awareness and training work to be done with physicians, mainly at the primary care level. Even with minor physical consequences, violence is always serious from the point of view of victims’ health and safety, as well as their families, and can be fatal. Thus, the complexities inherent in these cases cannot be a justification for lack of intervention [16].

### 4.6. Study Limitations and Further Research

The main limitation of the study is the bias related to the under-identification and/or underreporting of suspected cases of IPV. This was foreseen, as it is a known health condition throughout the world. We admit that there could exist additional information, but it is confidential and not possible to access. The obtained results did not allow us a differential diagnosis of the medicolegal aetiology of injuries and intoxications, and this aspect would be relevant to drawing more and better conclusions about these variables. Although we included a large female population and 20 years of retrospective analysis, only a descriptive study was performed at this stage. In future studies, we intend to include more health care units in Portugal and involve research groups form other European countries in order to perform more robust statistical analysis to compare variables.

## 5. Conclusions

Considering the obtained results, based on a real-life study, the following main conclusions can be highlighted:

1. At LHUM, only 2.3% (n = 1676) of women from 16 to 60 years old (n = 72,376) were identified and registered as suspected victims of IPV, which reflects an under-identification and/or underreporting situation; others may have been identified, with the suspicion in a confidential register for reasons of medical privacy;
2. At LHUM, physicians more often refer to the alleged IPV cases using clinical codes than clinical notes;
3. Women suspected of being victims of IPV are, in fact, people with more health problems than the general population of the same sex and geographic area. Comparing women who are suspected victims of IPV with the general population of women, the former present greater
  (a) Substance consumption (tobacco, alcohol, and drugs; 1.5, 6.5, and 13 times higher, respectively);
  (b) Traumatic injuries, particularly superficial injuries (2.2 times higher);
  (c) Intoxications (2 times higher);
  (d) Mental health disorders (namely sleep disorders, unspecified chronic pain, anxiety disorders, and major psychiatric disorders; 3.6, 2.4, 2.7, and 2.2 times higher, respectively);
  (e) Medication consumption (specifically anxiolytics, sedatives, antidepressants, and antipsychotics; 1.7, 2.0, 2.1, and 3.2 times higher, respectively);
  (f) Suicidal ideation (8.6 times higher);
  (g) Social deprivation (7.9 times higher);
  (h) Somatic diseases and other health conditions, such as obesity, metabolic syndrome, type 2 diabetes, hypertension, early heart disease, myocardial infarction, ischaemic and haemorrhagic stroke, urinary tract infection, sexually transmitted infections, asthma, and cancer, among others.

The under-detection and underreporting of suspected IPV cases by physicians are a reason for great concern and consideration, given the serious consequences that these cases represent for the health of victims.

## Figures and Tables

**Table 1:** Female population observed at LHUM, 2001–2021.

General Female Population of LHUM (n)	Suspected Female Victims of IPV—n (%)		
	G1 (Codes and Clinical Notes)	G2 (Codes)	G3 (Clinical Notes)
72,376	1676 (2.3)	766 (1.1)	931 (1.3)

**Table 2:** Victims’ history of substance consumption (health risk behaviours).

Substance Consumption	General Female Population		Suspected Female Victims of IPV					
			G1		G2		G3	
	n	%	n	%	n	%	n	%
Tobacco	12,356	17.1	395	23.6	200	26.1	199	21.4
Alcohol	274	0.4	22	1.3	19	2.5	4	0.4
Drugs	54	0.1	8	0.5	7	0.9	1	0.1

**Table 3:** Victims’ history of traumatic injuries and intoxications (medicolegal aetiology unknown).

Traumatic Injuries and Intoxications	General Female Population		Suspected Female Victims of IPV					
			G1		G2		G3	
	n	%	n	%	n	%	n	%
Bone fracture	23,998	33.2	796	47.5	409	53.4	394	42.3
Bone dislocation	1828	2.5	64	3.8	31	4.1	35	3.8
Open wound	17,835	24.6	621	37.1	307	40.1	322	34.6
Superficial injury	1850	2.6	76	4.5	43	5.6	35	3.8
Crushing injury	401	0.6	17	1	6	0.8	11	1.2
Burns	2161	3	76	4.5	40	5.2	35	3.8
Intoxications	9737	13.5	408	24.3	206	26.9	200	21.5

**Table 4:** Victims’ mental health conditions.

Mental Health Conditions	General Female Population		Suspected Female Victims of IPV					
			G1		G2		G3	
	n	%	n	%	n	%	n	%
Headaches	1793	2.5	79	4.7	52	6.8	28	3.0
Sleep disorders	5016	6.9	285	17.0	191	24.9	98	10.5
Eating disorders	133	0.2	9	0.5	6	0.8	4	0.4
Poor health perception	1348	1.9	58	3.5	36	4.7	24	2.6
Unspecified chronic pain	6947	9.6	286	17.1	179	23.4	114	12.2
Memory disorders	252	0.4	18	1.1	12	1.6	6	0.6
Anxiety disorders	15,552	21.5	699	41.7	440	57.4	271	29.1
Major psychiatric disorder	25,352	35.0	1091	65.1	579	75.6	527	56.6
Posttraumatic stress disorder	74	0.1	13	0.8	10	1.3	3	0.3
Suicidal ideation	212	0.3	36	2.2	19	2.5	19	2.0
Social deprivation	957	1.3	109	6.5	80	10.4	29	3.1
Anxiolytics consumption	37,263	51.5	1276	76.1	670	87.5	627	67.4
Sedatives consumption	18,583	25.7	727	43.4	392	51.2	341	36.6
Antidepressants consumption	28,080	38.8	1156	69.0	624	81.5	551	59.2
Antipsychotics consumption	6209	8.6	418	24.9	208	27.2	217	23.3

**Table 5:** Victims’ somatic conditions.

Somatic Conditions	General Female Population		Suspected Female Victims of IPV					
			G1		G2		G3	
	n	%	n	%	n	%	n	%
Obesity	12,570	17.3	389	23.2	234	30.5	162	17.4
Hypercholesterolemia	27,750	38.3	772	46.0	382	49.8	398	42.7
Metabolic syndrome	44,298	61.2	1 232	73.5	623	81.3	619	66.4
Type 2 diabetes	3354	4.6	157	9.3	92	12.0	69	7.4
Non-alcoholic fatty liver disease	436	0.6	35	2.0	17	2.2	19	2.0
Chronic obstructive pulmonary disease	416	0.6	17	1.0	8	1.0	9	1.0
Hypertension	13,262	18.3	421	25.1	267	34.9	163	17.5
Early heart disease	254	0.4	12	0.7	5	0.7	7	0.8
Myocardial infarction	212	0.3	11	0.7	5	0.7	6	0.6
Ischaemic stroke	893	1.2	55	3.3	30	3.9	26	2.8
Haemorrhagic stroke	63	0.1	4	0.2	3	0.4	1	0.1
Chronic kidney disease	544	0.8	24	1.4	10	1.3	15	1.6
Inflammatory pelvic disease	210	0.3	10	0.6	4	0.5	6	0.6
Urinary tract infection	3709	5.1	122	7.3	64	8.4	60	6.4
Sexually transmitted infections	1106	1.5	57	3.4	37	4.8	20	2.2
Preeclampsia	592	0.8	20	1.2	15	2.0	5	0.5
Desired pregnancy	1688	2.3	31	1.9	15	2.0	16	1.7
Undesired pregnancy	190	0.3	8	0.5	7	0.9	1	0.1
Spontaneous abortion	1767	2.4	82	4.9	45	5.9	38	4.1
Voluntary abortion	609	0.8	25	1.5	16	2.1	9	1.0
Chronic immune infective disorder	1085	1.5	32	1.9	15	2.0	17	1.8
Asthma	4583	6.3	141	8.4	72	9.4	70	7.5
Cancer	7550	10.4	253	15.1	124	16.2	131	14.1
Cervical cancer	248	0.3	10	0.6	6	0.8	4	0.4

## Data Availability

All aggregated statistical results are incorporated into this article. Patient level data used in this study is not publicly available.
